# Can salivary creatinine and urea levels be used to diagnose chronic kidney disease in children as accurately as serum creatinine and urea levels? A case–control study

**DOI:** 10.1080/0886022X.2017.1308256

**Published:** 2017-04-04

**Authors:** Rahime Renda

**Affiliations:** Department of Pediatric Nephrology, Antalya Research and Education Hospital, Antalya, Turkey

**Keywords:** Serum, saliva, creatinine, chronic kidney disease

## Abstract

**Background and aim:** Children with chronic kidney disease (CKD) develop many metabolic changes in blood that often necessitate frequent biochemical analysis. Serum analysis is an invasive and painful procedure. It would be highly beneficial if a noninvasive alternative process to serum analysis in children were identified. Saliva can be collected noninvasively, repeatedly, and without the use of healthcare personnel. The aims of this study were to compare serum and salivary urea and creatinine levels in children with CKD and healthy controls, and to determine if salivary creatinine and urea levels can be used to diagnose CKD in children as accurately as serum creatinine and urea levels.

**Materials and methods:** This case–control study included 35 children with CKD and 28 healthy children as controls. Saliva and blood samples were collected for measurement of urea and creatinine levels. The urea and creatinine levels in serum and saliva in the CKD and control groups were compared using the independent samples Mann–Whitney *U* test. Correlations between the serum and salivary urea and creatinine levels were determined using Pearson’s correlation coefficient. Receiver operating characteristic analysis was used to assess the diagnostic performance of salivary creatinine and cutoff values were identified.

**Results:** In the CKD group, the mean salivary creatinine level was 0.45 mg/dL and the mean salivary urea level was 0.11 mg/dL, versus 28.83 mg/dL and 21.78 mg/dL, respectively, in the control group. Stage 4 and 5 CKD patients had a mean salivary urea level of 31.35 mg/dL, as compared to 17.78 mg/dL in the control group. Serum urea and creatinine, and salivary creatinine were significantly higher in the CKD patients (regardless of disease stage) than in the controls (*p* < .05). The salivary urea level was significantly higher in the stage 4 and 5 CKD patients than in the controls (*p* < .05). There was a positive correlation between serum and salivary creatinine. The area under the curve for salivary creatinine was 0.805. The cutoff value for salivary creatinine was 0.125 mg/dL, with a sensitivity of 82.9% and specificity of 78.6%.

**Conclusions:** Based on the positive correlation between the serum and saliva creatinine levels observed in the present study, we think saliva analysis could be used as a noninvasive alternative to blood analysis for diagnosing CKD in children.

## Introduction

Chronic kidney disease (CKD) is characterized by high morbidity and mortality rates. The number of CKD patients under follow-up is rapidly increasing, especially children.^1^ The serum level of creatinine (primarily secreted by the kidneys) is used to determine renal function.^2^ CKD is multi-organ in origin and is associated with an increase in metabolic waste products in blood. Patients with CKD require frequent serum analysis to diagnose and monitor therapeutic results. Collection of blood for serum analysis is an invasive procedure associated with fear and anxiety in children. Frequent blood sampling results in severe anemia and an increase in the risk of infection;^3^ therefore, a simple noninvasive diagnostic test with minimal risk that can accurately evaluate disease status would be of tremendous value to patients and clinicians.

Saliva, a multi-constituent biologic fluid secreted by the salivary glands, plays an important role in oral and systemic health. Its collection for biochemical analysis is preferable to collection of blood because it is noninvasive, simple, and inexpensive, and can be performed more frequently. It also provides a cost-effective method for screening large populations.^4^^,^^5^ Numerous studies have reported that some systemic diseases, including CKD, produce significant detectable changes in saliva.^6^^,^^7^ Analysis of salivary creatinine and urea in CKD patients offers many advantages that have been attributed to the use of it as a diagnostic biofluid. Saliva can be affected by many factors and, as such, its use as a diagnostic fluid continues to be studied. The present study aimed to compare serum and salivary urea and creatinine levels in children with CKD and healthy controls, and to determine if salivary creatinine and urea levels can be used to diagnose CKD in children as accurately as serum creatinine and urea levels.

## Materials and methods

The study included 35 patients diagnosed as CKD, and 28 age- and gender-matched controls with a negative history of history of kidney and systemic disease. The study protocol was approved by the Antalya Research and Training Hospital Research Ethics Committee. The participants and their parents were provided information about study and parent’s verbal consent was obtained. Prior to collecting saliva all the participants underwent clinical examination of the oral cavity.

Based on the National Kidney Foundation Kidney Disease Outcome Quality Initiative (NFK KDOQI) classification system, CKD was diagnosed based on the presence of kidney damage for ≥3 months characterized by structural or functional abnormalities of the kidneys, with or without a decrease in the glomerular filtration rate (GFR), as evidenced by ≥1 of the following features: (a) abnormalities in blood or urine, (b) abnormal imaging findings, and (c) abnormal kidney biopsy findings; or a GFR <60 mL/min/1.73 m^2^ for ≥3 months.^8^ Stage 1 and 2 CKD were defined as a normal GFR or GFR >90 mL/min/1.73 m^2^ and kidney damage with a GFR 60–89 mL/min/1.73 m^2^, respectively. Stage 3, 4, and 5 CKD were defined as a GFR of 30–59, 15–29, and <15 mL/min/1.73 m^2^, respectively. Estimation of GFR was calculated using Schwartz’s formula.^9^ Serum and salivary creatinine were measured via the Jaffe method.^10^

Saliva was collected between 09:00 and 16:00, following fasting for ≥2 h. In CKD patients undergoing hemodialysis saliva was collected prior to dialysis. Whole saliva samples were collected via the spitting method. After rinsing the mouth with distilled water, each participant spit into a calibrated universal plastic bottle every 60 s until about 3 mL of saliva was obtained. Saliva samples were stored at −20 °C until laboratory analysis. Samples were thawed at room temperature and then centrifuged at 3000 rpm for 10 min in order to remove contaminants before analysis. Simultaneously, 5 mL of blood from each patient and control was drawn into a BD Vacutainer (Becton Dickinson, Franklin Lakes, NJ) SSTTM II Advance tube. The tubes were coated with micronized silica particles that activate clotting. Samples were checked for hemolysis or other interfering substances. Serum was then separated from the cells via centrifugation at 3000 rpm for 10 min.

### Analysis of plasma and salivary creatinine and urea

Serum and salivary creatinine levels were measured using a modification of Jaffe’s method,^10^ and urea levels were estimated using the urease method.^11^ Measurement was performed using commercially available kits (Beckman Coulter Diagnostics) and an autoanalyzer (Beckman AU 5800, Beckman Coulter Inc., Brea, CA). These methods have been used for saliva in earlier studies.^12^^,^^13^

### Statistical analysis

Data were recorded in a Microsoft Excel spreadsheet and were analyzed using SPSS for Windows v.17.0 (SPSS, Inc., Chicago, IL). Descriptive statistics, such as the mean, range, and standard deviation, were used to describe the main variables. The Kolmogorov–Smirnov test was conducted to assess the distribution of the variables in order to use a parametric or non-parametric test. Since all of our variables were normally distributed, parametric tests were employed. Correlations between plasma and salivary creatinine levels were determined using Pearson’s correlation analysis. Liner regression equations were derived to evaluate the serum level of creatinine from the salivary level. Receiver operating characteristic (ROC) analysis was performed to determine the diagnostic value of salivary creatinine, as compared to the serum level, and to correctly separate the CKD patients from the healthy controls. Overall performance was determined by the total area under the curve (AUC) and cutoff values were assessed based on the best tradeoff between sensitivity and specificity. The level of statistical significance was set at *p* < .05.

## Results

The CKD group included 35 children with CKD and the control group included 28 healthy children. Based on the estimated GFR, 7 (20%) patients were classified as stage 2 CKD, 8 (22.8%) as stage 3 CKD, 10 (28.6%) as stage 4 CKD, and 10 (28.6%) as stage 5 CKD. Among the 10 stage 5 CKD patients, 7 were undergoing hemodialysis and 3 were undergoing peritoneal dialysis, in addition to medical treatment. Patient and control demographics are shown in Table 1.

**Table 1.** Demographic parameters in the CKD and control groups.

	CKD group (*n* = 35)	Control group (*n* = 28)
Age (years)	14.49 ± 3.15 (range: 6–18)	14.86 ± 3.11 (range: 7–17)
Male (*n*)	18	16
Female (*n*)	17	12

Serum and salivary creatinine and urea levels in both groups are shown in Table 2. The mean serum and salivary creatinine levels were significantly higher in the CKD group than in the control group (*p* < .05). The mean serum urea level was significantly higher in the CKD group than in the control group (*p* < .05), whereas the mean salivary urea level was not significantly higher in the CKD group than in the control group (Table 2). Mean serum urea and creatinine levels in the stage 4 and 5 CKD patients (*n* = 20) were higher than in the stage 2 and 3 CKD patients. That’s why in these patients (stage 4 and 5 CKD) between control group (16 patients; age and gender matched), there was a statistically significant differences in mean salivary urea and creatinine (Table 3).

**Table 2.** Serum and salivary creatinine and urea levels in the CKD and control groups.

	Group	*n*	Mean	SD	*t*	*p*
Serum urea (mg/dL)	CKD	35	40.6286	25.01421	4.652	.000*
	Control	28	9.2857	2.23361		
Serum creatinine (mg/dL)	CKD	35	4.1409	4.28249	2.989	.004*
	Control	28	0.6971	0.15504		
Salivary urea (mg/dL)	CKD	35	28.8286	15.75452	1.516	.136
	Control	28	21.7857	11.44336		
Salivary creatinine (mg/dL)	CKD	35	0.4489	0.57186	2.208	.042*
	Control	28	0.1114	0.13114		

* Statistically significant.

**Table 3.** Serum and salivary creatinine and urea levels in the CKD stage 4 and stage 5 patients, and controls.

	Group	*N*	Mean	SD	*t*	*p*
Serum urea (mg/dL)	Stage 4–5 CKD	20	55.6000	23.05006	7.459	.000*
	Control	16	9.2857	2.23361		
Serum creatinine (mg/dL)	Stage 4–5 CKD	20	6.4220	4.45479	4.784	.000*
	Control	16	0.6971	0.15504		
Salivary urea (mg/dL)	Stage 4–5 CKD	20	31.3500	16.29102	2.191	.024*
	Control	16	17.7857	11.44336		
Salivary creatinine (mg/dL)	Stage 4–5 CKD	20	0.6535	0.68844	2.672	.012*
	Control	16	0.1514	0.15114		

* Statistically significant.

**Figure 1.**
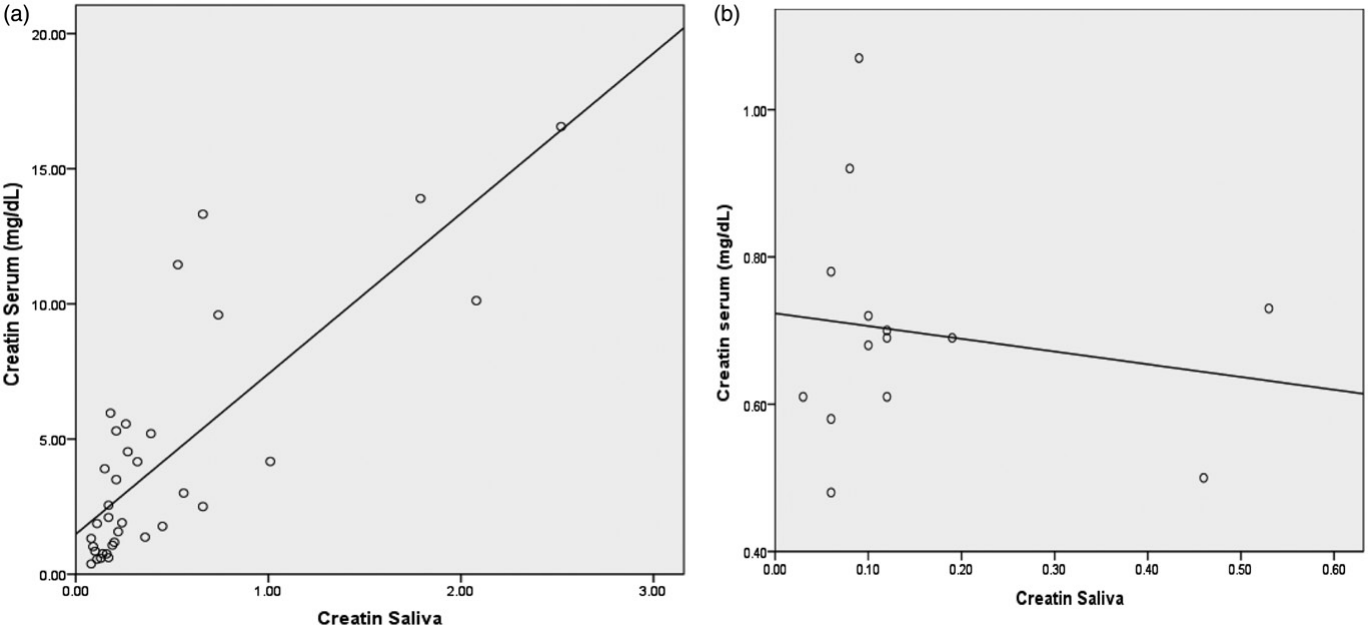
(a) Linear correlation between the salivary serum and creatinine levels in the CKD group. (b) Linear correlation between the salivary serum and creatinine levels in the control group.

To know if there was any association between serum and salivary creatinine and if changes in serum creatinine are caused changes in salivary creatinine, we achieved a correlation analysis of cases and control group. There was a significant positive correlation between the serum and salivary creatinine levels (Table 4). Linear regression analysis was performed to estimate the serum and salivary creatinine levels in both groups, which showed that there was a linear correlation between the salivary serum and creatinine levels (Figure 1(a,b)).

**Table 4.** The correlation between serum and salivary creatinine in the CKD and control groups, based on Pearson’s estimation.

	*r*	*p*
CKD group	0.792	.046*
Control group	−0.169	.012*

* Statistical significance (*p* < .05).

To determine the diagnostic utility of saliva, as compared to serum creatinine, that is, to correctly separate the group being tested into CKD patients and controls, ROC analysis was performed. The total AUC was 0.885 for serum creatinine and 0.805 for salivary creatinine (Table 5). The sensitivity and specificity for different levels of salivary creatinine were measured and a cutoff value of 0.125 mg/dL was estimated, as this yielded the best tradeoff with a sensitivity of 82.9% and specificity of 78.6% (Table 6).

**Table 5.** Area under the ROC curve.

	Area	Standard error	Asymptotic SIG.	Asymptotic 5% CI	
				Lower bound	Upper bound
Serum creatinine	0.885	0.048	0.000**	0.790	0.980
Salivary creatinine	0.805	0.075	0.001**	0.659	0.952

CI: confidence interval.

** Statistical significance (*p* < .05).

**Table 6.** Sensitivity and specificity analysis for salivary creatinine at different cutoff levels considered the gold standard.

Salivary creatinine (mg/dL)	Sensitivity (%)	Specificity (%)
0.045	100.0	7.10
0.105	88.6	57.1
**0.125**	**82.9**	**78.6**
0.205	54.3	85.7
0.295	37.1	85.7
0.495	25.7	92.9
0.545	22.9	100.0

## Discussion

Creatinine is a waste product of metabolism primarily excreted by the kidneys. All creatinine is excreted without reabsorption; as such, its levels in bloodare used as an index of kidney function.^2^ Due to an increase in serum creatinine and urea in children with CKD, the salivary creatinine and urea levels also increase because when there is renal failure the kidneys cannot excrete creatinine and its blood level increases.^2^ Elevated salivary creatinine and urea causes dry mouth,^14^ uremic breath,^15^ tongue coating, and other oral complications^16^ of CKD. A high concentration of urea and creatinine in saliva might be due to elevated serum creatinine and urea levels, which produce and elevated level gradient which in turn increases the diffusion of creatinine and urea from serum to saliva in CKD patients.^17^ It could be that saliva is an alternative route of excretion when renal function is impaired.

The present findings show that children with CKD (stage 2–5) have higher levels of salivary creatinine than healthy children. The present study’s stage 4 and 5 CKD patients had higher levels of serum urea and creatinine than those with stage 2 and 3 disease; therefore, the stage 4 and 5 CKD patients had significantly higher salivary creatinine and urea levels than the controls. The salivary urea concentration is associated with the severity of kidney disease, especially in dialyzed patients.^12^^,^^18^ The positive correlation between serum and salivary creatinine as well as urea consistent with previous reports.^19–21^ The literature includes only a few studies on children with renal disease and saliva contents. Among the aims of the present study were to determine if saliva could be used to diagnosis CKD in children.

Creatinine is a large molecule with a high molecular weight that exhibits low lipid solubility. In healthy individuals, it is unable to diffuse across the cells and the tight junction of the salivary gland,^2^ but in CKD patients serum creatinine increases, a concentration gradient occurs, and creatinine diffusion increases from serum to saliva.^17^ In the present study, there was a positive correlation between serum and salivary creatinine in the CKD group and a negative correlation in the control group, as reported earlier in adult patients with CKD.^22^^,^^23^ To determine the association between serum and salivary creatinine levels in the present study linear regression analysis was performed, which showed that there was a linear correlation between salivary and serum and creatinine levels in the CKD group and control group, as reported by Venkatapathy et al.^22^

Before salivary testing of urea and creatinine can be adopted as a diagnostic method to replace more conventional methods, the diagnostic value of salivary testing must be compared to that of accepted standard methods.^24^ The accuracy of the new test depends on how well it separates the group being tested into those with and without the disease.^25^ Sensitivity and specificity are basic indexes used to determine the accuracy of any diagnostic test; therefore, ROC analysis is used to understand the diagnostic potential of saliva as an alternative to a standard method. Accuracy is estimated by the area under the ROC curve (AUC). In the present study, the AUC was 0.805, which indicates that the salivary creatinine level is a good alternative diagnostic test for differentiating children with CKD children and healthy children. Study reports included adult CKD patients found similar rates of AUC, 0.897^26^ and 0.967,^22^ respectively. Using ROC analysis in the present study multiple salivary creatinine cutoff levels for diagnosing kidney disease were obtained; a cutoff level of 0.125 mg/dL had sensitivity of 82.9% and specificity of 78.6%, indicating that children with a salivary creatinine level >0.125 mg/dL are more likely to have CKD and require further evaluation for appropriate management. The present findings suggest that saliva can be used as an alternative biofluid for estimating serum creatinine in children with CKD.

## Conclusions

The present study’s findings show that the salivary creatinine level in children with stage 2–5 CKD and the salivary urea level in those with stage 4 and 5 CKD were positively correlated to their serum levels. ROC analysis showed good sensitivity and specificity levels for salivary creatinine. Based on these findings, we think that saliva could be an alternative to blood for diagnosis and monitoring children with CKD. Saliva collection is a noninvasive method for obtaining diagnostic fluid in patients with CKD, and can reduce the anxiety and discomfort associated with blood collection, can be taken to allow frequent that will increase the monitor these patients general health over time to diagnose morbidities in the early stages. The most important finding in the present study is that saliva can be used as a noninvasive diagnostic tool for estimating the serum creatinine level in children with CKD. Unlike other studies, our study comprised children in all the stages of CKD and healthy controls. Additional larger scale controlled studies in children with CKD are needed to further understand the role of saliva analysis in the diagnosis and treatment.
